# Open Surgical Repair of Proximal Hamstring Tears Results in High Rates of Hamstring Strength Recovery and Return to Preinjury Activity

**DOI:** 10.1002/ars2.70150

**Published:** 2026-10-21

**Authors:** Mohammad Mesfer Alzahrani, Brendon Swift, Zaid A. Alzaid, Abdulhamid Alyousef, Michael Pickell, Geoffrey P. Wilkin, Steven R. Papp, Allan S. Liew

**Affiliations:** ^1^ Department of Orthopaedic Surgery, College of Medicine Imam Abdulrahman Bin Faisal University Dammam Saudi Arabia; ^2^ Division of Orthopaedic Surgery, University of Ottawa The Ottawa Hospital Ottawa Ontario Canada; ^3^ Department of Orthopaedic Surgery King Fahad Hospital Al Hofuf Saudi Arabia

## Abstract

**Purpose:**

To evaluate the clinical and patient‐reported outcomes after open operative management of proximal hamstring ruptures.

**Methods:**

Retrospective chart review was completed for patients who underwent open surgical repair of a proximal hamstring rupture at a single academic center (The Ottawa Hospital) with a minimum of 1‐year follow‐up between June 2017 and August 2022. Demographic, injury, and surgical data were collected. Patient‐reported outcomes, including the Lower Extremity Function Score and International Hip Outcome Tool, at final follow‐up were collected and analyzed for all patients.

**Results:**

A total of 41 patients (58.5% men) with a mean age of 49.5 ± 11.7 years (range: 20‐70 years old) with a mean follow‐up of 46.8 ± 17.6 months (range 13‐74 months) were included in the study. The most common mechanism of injury (31.7%) was a fall while skating or skiing. A 3‐tendon injury was the most common pattern (82.9%), with the majority being retracted at least 6 cm (51.2%). Postoperative complications were observed in 12.2% of patients and included sensory nerve injury, repair failure, and postoperative admission for intractable pain. Most patients regained full hamstring strength (87.8%) and returned to preinjury activity levels (70.7%), with a mean follow‐up of 46.8 months. Patient‐reported outcomes at final follow‐up were excellent, as 87.8% of the patients scored >90 on Lower Extremity Function Score and 82.9% scored >90 on International Hip Outcome Tool.

**Conclusions:**

In this study, patients who underwent open surgical repair of proximal hamstring tendon avulsion had high rates of hamstring strength recovery and return to preinjury activity, with favorable patient‐reported outcomes and low complication rates.

**Level of Evidence:**

Level IV, therapeutic retrospective study.

## Graphical abstract

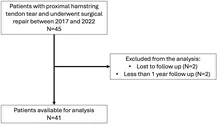

Hamstring injuries commonly affect athletes and active individuals. Proximal hamstring tendon avulsions frequently occur from eccentric contraction with the hip flexed and knee extended, placing the muscle at or near its maximum length and exposing it to risk of injury. Much of the epidemiological data available arises from professional sports registries.^1^, ^2^, ^3^, ^4^ Although this provides useful information in the pathomechanics of injury in high‐level athletes, it can still aid in informing management in the general population. However, additional factors should be considered in the average patient as the risk of hamstring injury increases with age, decreased hip flexibility, and hamstring weakness relative to quadriceps.^5^, ^6^, ^7^

Commonly recognized indications for surgical repair include 2 or more tendon involvement with 2 or more centimeters of retraction.^8^, ^9^, ^10^ Conversely, patient comorbidities and overall level of activity must be taken into consideration because nonoperative management may be selected in elderly, comorbid, or inactive patients unwilling to participate in the required rehabilitation. Although nonoperative management of proximal hamstring avulsions avoids operative complication risk, studies have shown marked functional deficits and lower patient‐reported outcomes in patients with nonoperatively managed complete tendon avulsions as compared with the uninvolved limb.^11^, ^12^, ^13^ The purpose of this study was to evaluate the clinical and patient‐reported outcomes after open operative management of proximal hamstring ruptures. We hypothesized that surgical repair would result in favorable patient‐reported outcomes, a high rate of return to preinjury activity, and an acceptable complication profile.

## METHODS

This is a single‐institution retrospective study of patients who sustained an acute proximal hamstring tendon tear and underwent surgical repair between June 2017 and August 2022. Institutional Review Board approval was obtained from The Ottawa Hospital, Ottawa, ON, Canada (Protocol ID#: 20230024‐01H) to conduct this study. Inclusion criteria were skeletally mature patients with a history of proximal hamstring tendon tear, confirmed by ultrasound or magnetic resonance imaging performed at the same center and interpreted by a musculoskeletal‐trained radiologist (classified according to the British Athletics Muscle Injury Classification), and repaired surgically with a minimum follow‐up of 1 year. Patients with ipsilateral lower extremity skeletal injury, patients with a tear in the muscle belly, patients with preoperative sciatic nerve symptoms, patients lost to follow‐up, and patients with less than 1‐year follow‐up were excluded from the study.

### Surgical Technique

Patients diagnosed with acute proximal hamstring tendon tears who opted for surgical management had an open proximal hamstring tendon repair. The surgery was performed under general or spinal anesthesia in the prone position. All bony prominences were padded and preoperative antibiotics administered. According to the surgeon's preference, a horizontal or longitudinal incision was performed at the level of the gluteal crease centered over the palpated ischium. Dissection was taken down through the skin and subcutaneous tissue down to the posterior fascia, which is incised, taking care to protect the sciatic nerve (lateral to the torn tendons), which is kept protected during the length of the procedure, after which the torn tendon ends are identified and delivered through the incision. The tendon ends are then prepared and mobilized, and nonabsorbable thick running sutures are applied. The ischial attachment is then localized, and using a curette or rasp, the bony bed is freshened. Then, 1 to 4 anchors are applied to the bony bed with a 3‐mm separation (number of anchors is according to surgeon preference and bony bed size). The tendons are then tied to the anchors with adequate tension. The wound is then irrigated and closed in a layered fashion.

### Postoperative Protocol

Postoperative patients are allowed toe‐touch weight‐bearing with a full extension knee brace for 6 weeks. Trunk and hip flexion are restricted during this time. Patients are seen in clinic at 2 weeks postoperatively for a surgical wound check and suture removal and then referred for physical therapy.

Patient demographics were collected, including patient age, gender, side, and mechanism of injury. Injury data included the number of tendons injured and distance of retraction. Surgical data collected included the time elapsed from injury to surgery, type of anesthesia, type of incision, type of fixation used, and occurrence of any intraoperative complications. Duration of follow‐up, complications, hamstring strength as measured by the orthopaedic surgeons (M.M.A., M.P., G.P.W., S.R.P., A.S.L.) using motor grade on physical examination (Medical Research Council Scale)^14^ compared with the contralateral normal side, and self‐reported return to preinjury level of activity were also documented at 1‐year follow‐up. Patient‐reported outcome measures, including Lower Extremity Functional Score (LEFS), International Hip Outcome Tool, and visual analog scale pain score, were assessed at final follow‐up.

### Statistical Analysis

Continuous data was summarized using mean ± standard deviation (SD) or median with interquartile ranges. Categorical variables were summarized using frequency counts and percentages. Statistical analysis was performed using *χ* ^2^ testing for categorical data, whereas comparison of continuous variables was conducted using Student's *t* test or Mann‐Whitney *U* test for parametric and nonparametric distributions, respectively. Results were considered statistically significant if *P* < .05.

## RESULTS

A total of 41 patients were included in the study with a mean age of 49.5 ± 11.7 years (20‐70 years old), with a follow‐up rate of 91.1% and a mean follow‐up of 46.8 ± 17.6 months (range 13‐74 months) (Figure 1). Of the cohort, 58.5% were men, with most having left‐sided injuries (61%) (Table 1). The most common mechanism of injury was skating/skiing (31.7%), followed by slip and sports injuries (29.3% and 22%, respectively) (Table 1). Magnetic resonance imaging was the modality of choice for diagnosis in 75.6% of cases. A 3‐tendon injury was most commonly identified (82.9%), and 51.2% were retracted at least 6 cm (range of cohort retraction 2‐22 cm). A significant association was found between the number of tendons injured and patient age, as single‐tendon injury mean age was significantly less than that of 2‐ and 3‐tendon injuries (30.7 ± 13.4, 49 ± 18.5 and 51.2 ± 9.4, respectively, *P* value = .01, effect size [eta‐squared] = 0.21).

**FIGURE 1:**
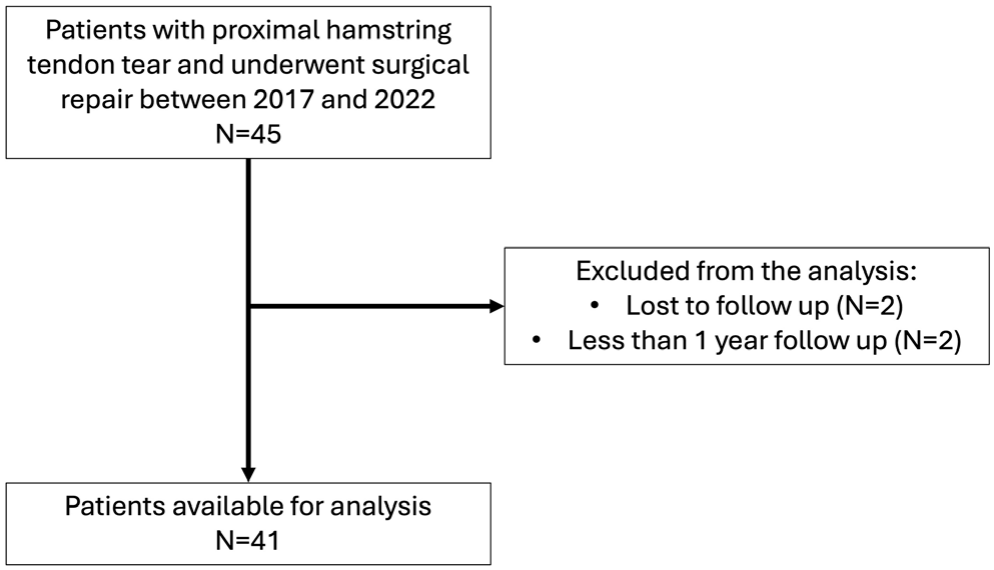
Study patient flowchart.

**TABLE 1:** Demographic and Postoperative Data

**Variables**		**N (Percentage)**
Sex	Male	24 (58.5%)
	Female	17 (41.5%)
Mechanism of injury	Skating/skiing	13 (31.7%)
	Slip	12 (29.3%)
	Sports	9 (22%)
	Running	4 (9.8)
	Other sports activity	3 (7.3%)
Side	Left	25 (61%)
	Right	16 (39%)
Imaging	MRI	31 (75.6%)
	US	10 (24.4%)
Tendons injured	1	3 (7.3%)
	2	4 (9.8%)
	3	34 (82.9%)
Retraction, cm	<6	20 (48.8%)
	≥6	21 (51.2%)
Anesthesia	General	39 (95.1%)
	Spinal	2 (4.9%)
Incision	Horizontal	22 (53.7%)
	Longitudinal	19 (46.3%)
Fixation	Anchors	39 (95.1%)
	Other (screws, suture repair)	2 (4.9%)
Fixation number	1	1 (2.6%)
	2	12 (30.8%)
	3	18 (46.2%)
	4	8 (20.5%)
Complications	Yes	5 (12.2%)
	No	36 (87.8%)
Hamstring strength	Full	36 (87.8%)
	Partial	5 (12.2%)
Return to preinjury level of activity	Yes	29 (70.7%)
	No	12 (29.3%)

MRI, magnetic resonance imaging; US, ultrasound.

Concerning operative data, the median time from injury to surgery was 22 days (interquartile range 16.5‐35, range 7‐153 days). General anesthesia was the most common form of anesthetic used for the procedure (95.1%). Regarding surgical technique, a horizontal surgical incision was most commonly used (53.7%), employing suture anchor fixation (95.1%), most commonly using a 3‐anchor construct (46.2%) (Table 1).

Within the cohort, a complication rate of 12.2% was observed. This included 2 sensory nerve injuries (posterior cutaneous nerve of the thigh), both of which self‐resolved, 2 repair failures (because of reinjury), and 1 readmission for intractable postop pain that improved with pain medications and had no specific cause. This pain resolved at final follow‐up. Most patients regained full hamstring strength (87.8%) and returned to preinjury activity levels (70.7%). The number of anchors used for fixation was significantly associated with complications because using only 1 or 2 anchors had a higher rate of complications (both repair failures occurred in patients fixed with only 2 anchors) (Table 2). We found no significant association of complications, hamstring strength, and return to preinjury level of activity with remaining demographic and operative data.

**TABLE 2:** Patient Outcome Scores and Demographic and Operative Data

		**Complications**		**Hamstring Strength**		**Return to Preinjury Level of Activity**	
		**Yes (5)**	**No (36)**	**Full (N = 36)**	**Partial (N = 5)**	**Yes (29)**	**No (12)**
Age, yr		48.4 ± 6.02	49.6 ± 1.94	49.97 ± 11.76	45.8 ± 11.95	50.55 ± 11.58	46.83 ± 12.1
		NS		NS		NS	
Sex	Male	2	22	21	3	15	9
	Female	3	14	15	2	14	3
		NS		NS		NS	
Tendons injured	1	1	2	2	1	2	1
	2	0	4	4	0	3	1
	3	4	30	30	4	24	10
		NS		NS		NS	
Retraction	<6 cm	1	19	18	2	15	5
	≥6 cm	4	17	18	3	14	7
		NS		NS		NS	
Incision	Horizontal	3	19	21	1	15	7
	Longitudinal	2	17	15	4	14	5
		NS		NS		NS	
Time to surgery	<2 wk	2	6	7	1	6	2
	2‐4 wk	1	14	14	3	12	3
	>4 wk	2	16	15	3	11	7
		NS		NS		NS	
Fixation number	1	1	2	2	1	2	1
	2	3	9	11	1	7	5
	3	1	17	16	2	15	3
	4	0	8	7	1	5	3
		*P* < .05		NS		NS	

NS, not significant; wk, weeks; yr, years.

Patient‐reported outcomes were excellent at final follow‐up, as 87.8% of the patients scored >90 on the LEFS and 82.9% scored >90 on the International Hip Outcome Tool. Pain scores were also excellent at final follow‐up, as visual analog scale pain scores were 0 in 78% of patients, and all patients reported a visual analog scale pain score of ≤5 at final follow‐up. Patient‐reported outcomes were significantly greater in patients without complications, patients who returned to full hamstring strength, patients who returned to their preinjury baseline, and patients who were satisfied with their experience (Table 3).

**TABLE 3:** Patient Outcome Scores and Postoperative Outcomes

	**Complications**		**Hamstring Strength**		**Return to Preinjury Level of Activity**	
	**Yes (5)**	**No (36)**	**Full (N = 36)**	**Partial (N = 5)**	**Yes (29)**	**No (12)**
LEFS	63.3 ± 29.3	97.8 ± 6.3	97.1 ± 10.9	68.3 ± 23.7	98.8 ± 4.2	80.9 ± 25
	*P* < .001		*P* < .001		*P* < .001	
iHOT	62.7 ± 29.9	96.3 ± 10.2	96.7 ± 10.8	58.8 ± 21.2	98.6 ± 4.2	76.7 ± 26.3
	*P* < .001		*P* < .001		*P* < .001	

iHOT, International Hip Outcome Tool; LEFS, Lower Extremity Functional Score.

## DISCUSSION

The most important finding of this study was that the repair of proximal tendon avulsions is a viable treatment for the general population with favorable patient‐reported outcomes, with most patients (>80%) scoring more than 90 on the LEFS and the International Hip Outcome Tool. Our cohort also had a high rate of return to preinjury level of activity with an acceptably low complication rate. Similar to a study examining outcomes after proximal hamstring repair, as Hillier‐Smith et al. showed in their systematic review, surgically managed proximal hamstring avulsions had an 84.5% return to sport with good functional outcomes (mean LEFS of 74.7).^9^

Most patients (58.5%) in the cohort were men, with an average age of 49.5 years, consistent with a study of hamstring avulsion repair in a large‐scale patient cohort.^15^ Based on our region's winter activity profile, we observed a high frequency of skiing and skating injuries (31.7%), which understandably may not occur with the same frequency in different climates.^15^ When combined with other sports injuries, this constitutes 54% of the cohort, again similar to previous reports.^15^, ^16^

Most patients in the present series underwent open surgical repair for acute 3‐tendon injuries (82.9%). A much smaller proportion of patients were found to have a single‐ (7.3%) or 2‐tendon (9.8%) tear at the time of surgery, highlighting the fact that patients with higher‐grade injuries were those who were offered and ultimately underwent surgical repair. Surgical characteristics showed a slight surgeon preference toward a horizontal skin incision (53.7%), a strong preference for suture anchor‐based repair (95.1%), and most commonly repaired with a 3‐anchor construct (46.2%).

Furthermore, patients reported a high subjective rate of return to full hamstring strength (87.8%) and return to baseline level of function (70.7%). These findings are similar to published literature on surgical management of proximal hamstring injuries, as Bodendorfer et al. performed a systematic review comparing operative and nonoperative management of proximal hamstring tears. They found that the surgical cohort outperformed the nonsurgical group in all aspects, including hamstring strength (85.01% vs 63.95%).^12^

A recent international survey of medical professionals in orthopaedic traumatology and sports medicine emphasized that the decision for operative or nonoperative treatment of proximal hamstring avulsion should be based on the individual patient.^17^ The 5 most commonly identified decision modifiers they highlighted included presence of neurological symptoms, tendons involved, degree of tendon retraction, diminished level of patient function, and patient preference.^17^

### Limitations

The findings of the present study are taken along with a few limitations. Because this is a single‐center retrospective analysis of surgically treated cases, there exists a treatment bias and the inability to compare functional outcomes against similar patients treated nonoperatively. Notably, the absence of a comparison group undergoing nonoperative treatment of proximal hamstring prevents any comparison of differences in outcomes between operatively and nonoperatively managed patients. Given the unanticipated nature of hamstring injury in the general population, we do not have baseline preinjury patient reported outcome measures in this patient cohort to compare against. Furthermore, because of resource limitations, no biometric or sport‐specific return‐to‐activity testing was available for analysis, but rather, the return of strength and functioning was based on physical examination strength testing and patient subjective reporting. Also, an analysis of the data by sex distribution was not feasible because of the low number of patients.

## CONCLUSIONS

In this study, patients who underwent open surgical repair of proximal hamstring tendon avulsion had high rates of hamstring strength recovery and return to preinjury activity, with favorable patient‐reported outcomes and low complication rates.

## DISCLOSURES

The authors (M.M.A., B.S., Z.A.A., A.A., M.P., G.P.W., S.R.P., A.S.L.) declare that they have no known competing financial interests or personal relationships that could have appeared to influence the work reported in this paper.
